# Rhabdomyosarcome orbitaire chez un nourrisson de 9 mois

**DOI:** 10.11604/pamj.2019.34.192.4353

**Published:** 2019-12-11

**Authors:** Soumya Benziane, Rajae Daoudi

**Affiliations:** 1 Hôpital des Spécialités, Rabat, Maroc

**Keywords:** Rhabdomyosarcome orbitaire, prolifération tumorale, chimiothérapie, Orbital rhabdomyosarcoma, necrosed sarcomatous tumoral, chemotherapy

## Abstract

A 9-month-old baby, born within the normal period of gestation and with normal psychomotor development, presented with a rapidly progressive small swelling persisting for several months and evolving towards an inflammatory status. CT scan of the orbit and brain after contrast injection showed right intraorbital tissue mass forcing the eyeball upwards and outwards. The patient underwent surgical biopsy of the lesion. The diagnosis was necrosed sarcomatous tumoral proliferation with undifferentiated cells of small size; immuno-histochemical examination revealed embryonic rhabdomyosarcome. The patient was lost to follow-up for 3 months, then re-consulted. MRI evaluation was requested. This showed a significant increase in tumor volume. Initial chemotherapy was recommended followed by locoregional radiotherapy. The baby died a few months later.

## Image en médecine

Enfant de 9 mois né à terme d’une grossesse normale et de développement psychomoteur normal, a présenté depuis quelques mois une petite tuméfaction palpébrale supérieure rapidement progressive évoluant dans un contexte inflammatoire. Une tomodensitométrie (TDM) orbito-cérébrale après injection de produit de contraste note une masse tissulaire intra-orbitaire droite refoulant le globe oculaire en dehors et en haut. Une biopsie chirurgicale de la lésion a été réalisée, celle-ci fait état d’une prolifération tumorale sarcomateuse nécrosée faite de cellule indifférenciée de petite taille, l’étude immuno-histochimique a conclu à un rhabdomyosarcome de type embryonnaire. Le patient a été perdu de vue pendant 3 mois, puis à reconsulté. Une imagerie par résonance magnétique (IRM) d’évaluation a été demandée. Celle-ci a noté une importante progression du volume tumorale. Une chimiothérapie première a été préconisée puis radiothérapie locorégionale. L’enfant est décédé quelques mois plus tard.

**Figure 1:**
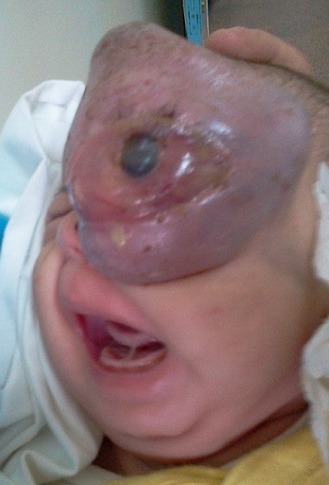
Rhabdomyosarcome orbitaire

